# Adults' Arithmetic Builds on Fast and Automatic Processing of Arabic Digits: Evidence from an Audiovisual Matching Paradigm

**DOI:** 10.1371/journal.pone.0087739

**Published:** 2014-02-05

**Authors:** Delphine Sasanguie, Bert Reynvoet

**Affiliations:** 1 Experimental Psychology, KU Leuven, Leuven, Belgium; 2 Faculty of Psychology and Educational Sciences @Kulak, Kortrijk, Belgium; The University of Western Ontario, Canada

## Abstract

Several studies have shown that performance on symbolic number tasks is related to individual differences in arithmetic. However, it is not clear which process is responsible for this association, i.e. fast, automatic processing of symbols per se or access to the underlying non-symbolic representation of the symbols. To dissociate between both options, adult participants performed an audiovisual matching paradigm. Auditory presented number words needed to be matched with either Arabic digits or dot patterns. The results revealed that a distance effect was present in the dots-number word matching task and absent in the digit-number word matching task. Crucially, only performance in the digit task contributed to the variance in arithmetical abilities. This led us to conclude that adults' arithmetic builds on the ability to quickly and automatically process Arabic digits, *without the underlying non-symbolic magnitude representation being activated*.

## Introduction

Over the last decade, studies investigating the relationship between individual differences in basic numerical abilities and mathematics achievement in both adults and children have multiplied. One of the crucial questions in this emerging body of research is whether processing magnitudes in either symbolic (e.g. Arabic digits) or non-symbolic (e.g. dot arrays) format is crucial to mathematics achievement [1]. Studies that assigned both symbolic and non-symbolic number tasks have shown that performance on *symbolic number tasks*, especially, is related to individual differences in mathematics achievement, in children [2], [3], [4] and in adults [5], [6].

Debate exists about what exactly in symbolic number processing is responsible for this association. The dominant interpretation for the relation between symbolic number processing and mathematics achievement is that strong mathematical competences originate with efficient *access* to the underlying innate non-symbolic representation when confronted with a symbol [7]. This claim stems from evidence that showed a correlation between the numerical distance effect (DE) observed in symbolic but not non-symbolic number processing tasks, and mathematics achievement [8], [9]. The DE [10] refers to the finding that people discriminate between two number stimuli more quickly and accurately as the distance between the two stimuli increases (e.g., 1–2 versus 1–9). The effect is explained by assuming overlapping representational distributions of numerical magnitudes on a ‘mental number line’ – the closer two magnitudes are on the line, the more overlap between their distributions and the harder to discriminate between them [11], [12]. Therefore, the DE is believed to reflect the activation of the underlying magnitude representation, with a larger DE indicating less precise or more noisy representations [13].

Alternatively, mathematics achievement may build on the *fast and automatic processing of symbols per se* (i.e. pure symbol processing without necessarily activating the non-symbolic magnitude representation). This hypothesis is in line with the observation that in several studies using symbolic number processing tasks, reaction times (RTs) correlated with mathematics achievement more than the DE, the latter being the reflection of magnitude activation [for an overview, see 1]. It has been suggested that, in addition to a non-symbolic magnitude representation, people might also have *a symbolic system* for processing numerical symbols that is exact in nature and emerges when children learn the meaning of number words [14], [15], [16], [17]. Evidence for such a distinct symbolic system has already been provided by behavioral studies with both children [18] and adults [19]. In 5-year-old children, Sasanguie et al. [18] showed that performance on a non-symbolic comparison task was not predictive of these children's performance on a symbolic comparison task measured six months later, suggesting no relation between non-symbolic and symbolic representations. In adults, it has been demonstrated that people are slower when they need to activate both symbolic and non-symbolic representations in the same task, possibly because of the additional processing cost it takes to connect both representations [19], [20]. Also neuroimaging studies have suggested that distinct neural processes are used for processing symbolic and non-symbolic magnitudes [21], [22]. Other studies [23] have extensively shown that for some arithmetic processes (e.g. retrieving the outcome of a multiplication problem) the main areas involved are those assumed to house verbal symbolic representations. Together, these results suggest that symbols may be processed in a separate, symbolic system that may serve as a basis for later arithmetic and is not necessarily connected to the non-symbolic magnitude representation.

**Figure 1:**
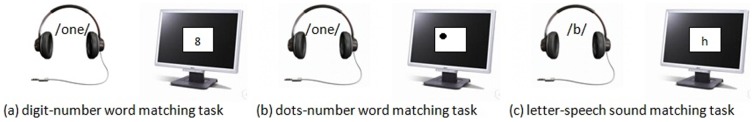
Illustration of the audiovisual matching paradigm used in the three experimental tasks. (a) the digit-number word matching task, (b) the dots-number word matching task and (c) the letter-speech sound matching task. In all three tasks, participants were simultaneously presented with an auditory stimulus (number or letter) and a visual stimulus (a digit, a dot pattern or a letter). They had to indicate whether what they heard and saw was ‘same’ or ‘different’.

To distinguish the hypothesis highlighting the importance of accessing the underlying non-symbolic magnitude from the hypothesis that arithmetical abilities build on the fast and automatic processing of symbols per se (i.e. without the underlying non-symbolic magnitude representation being activated), we used *an audiovisual matching paradigm.* Adult participants were presented with auditory number words that needed to be matched with either visually presented Arabic digits (i.e. pure symbolic task – see Figure 1a) or dot patterns (i.e. a mixed notation task – see Figure 1b). Their arithmetical abilities were tested with a standardized arithmetic test. Participants also completed a general processing speed task and an intellectual reasoning test. We reasoned that if individual differences in arithmetic are explained by efficiently *accessing* the underlying non-symbolic representation when confronted with a symbol, we would observe an association between performance on the mixed notation task – in which a symbolic number word needed to be matched with to a non-symbolic magnitude representation – and the arithmetic test score. On the other hand, if *fast and automatic processing of symbols per se* is responsible for individual differences in arithmetic, a relationship between the arithmetic test scores and performance on the pure symbolic task is to be expected. Here, in contrast to the mixed notation task, the task can be completed within the symbolic system. Moreover, to examine whether the relationship between arithmetic and symbol processing is number-specific, a third experimental task was included in which participants had to match an auditory presented speech sound to a visually presented letter (see Figure 1, c). If that relation was number-specific, no relationship between the latter pure symbolic task and arithmetical abilities should be witnessed.

## Methods

### Participants

Fifty-eight university students from the KU Leuven Kulak participated for eight euros. Six participants were excluded from the analyses because they performed too slow or made too many errors (>3*SD* above/below the group mean) in one of the experimental tasks. The final sample consequently consisted of 52 adults, *M*_age_ = 19.17 years; *SD* = 2.42; 29 females (SPSS file of the data available on request via email to the corresponding author).

### Ethics statement

Written informed consent was obtained following the Declaration of Helsinki, and this study was approved by the Ethical Committee of the KU Leuven.

### Procedure

Each participant first conducted the general processing speed task, followed by the experimental tasks (i.e. pure symbolic tasks: digit-number word matching and letter-speech sound matching; and the mixed notation task: dots-number word matching) in a fully counterbalanced way. Finally, the participants completed the standardized tests (arithmetic and intellectual reasoning).

#### Experimental tasks

*Digit-number word matching task:* Stimulus presentation and data recording were controlled by E-prime 1.1 (Psychology Software Tools, http://www.pstnet.com). Arabic digits were visually presented on the computer screen and the participants simultaneously heard a number word (e.g. ‘one’). The participants had to decide as quickly and accurately as possible whether the number word that they heard and the digit that they saw were either the same (a match) or different (no match) by pressing “a” or “p” on an AZERTY keyboard. Stimuli consisted of 1, 2, 8 and 9 in visual notation format (Arabic digits in white against a black background, Arial font 40) and auditory format (number words). The sounds were digitally recorded (sampling rate 44.1 kHz, 16-bit quantization) by a Dutch female speaker. Recordings were band-pass filtered (180–10.000 Hz), resampled at 22.05 kHz, and matched for loudness. The sounds were presented binaurally through loudspeakers at about 65 dB SPL. Each recorded number lasted about 1000 ms: [e.n] (one)  =  796 ms, [twe.] (two)  =  869 ms, [aχt] (eight)  =  716 ms, [ne.γ
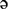
n] (nine)  =  997 ms. This way, 12 trials were obtained with an equal number of trials per condition: four same trials (i.e. 1-1, 2-2, 8-8 and 9-9), four different trials with a close numerical distance of one (i.e. 1-2, 2-1, 8-9 and 9-8), and four different trials with a far numerical distance of seven (i.e. 1-8, 8-1, 2-9 and 9-2). These 12 trials were randomly presented five times to the participants to obtain a final trial list of 60 trials. Before the start of the experiment start, five practice trials were given, during which feedback was provided to familiarize the participants with the task requirements. Each trial was preceded by a fixation cross of 600 ms and was presented for 1000 ms. Participants could respond during the stimulus presentation or during a blank screen that followed the stimulus presentation. The next trial began after an inter-trial interval of 1000 ms. Participants were seated at approximately 50 cm from the screen.

*Dots-number word matching task:* The procedure was identical to the digit-number word matching task, except that the stimuli were dot arrays with one, two, three [dri.] ( = 728 ms) or four [vi:r] ( =  807 ms) dots. Small numerosities from within the subitizing range (i.e. small number of up to 4 items that can readily be identified) [24] were chosen to enable rapid and exact identification of the presented magnitude. Stimuli were generated with the Matlab script developed by Gebuis and Reynvoet [25], controlling for four visual parameters: (1) the convex hull (i.e. smallest contour around the array of dots), (2) the aggregate surface of the dots, (3) density (i.e. the aggregate surface divided by the convex hull), and (4) the average diameter. Regression analyses confirmed that no relationship existed between most visual cues and numerosity (all *R^2^*s<.02, all *p*s >.15), except for aggregate surface (*R^2^*s = .11, *p*<.001). The trial list consisted of four same trials (i.e. 1-1, 2-2, 3-3 and 4-4), four different trials with a close numerical distance of one (i.e. 1-2, 2-1, 3-4 and 4-3), and four different trials with a far numerical distance of two (i.e. 1-3, 3-1, 2-4 and 4-2) – all presented randomly five times, resulting in a trial list of 60 trials.

*Letter-speech sound matching task:* The procedure was identical to the digit-number word matching task, with the exception that the stimuli were alphabet letters: [a.] (a, 607 ms), [be.] (b, 800 ms), [ha.] (h, 757 ms) and [i.] (i, 494 ms). As in the digit and the dot task, there was an equal number of trials per condition: four same trials (a-a, b-b, h-h and i-i), four different trials with a close numerical distance of one (a-b, b-a, h-i and i-h), and four different trials with a far numerical distance of seven (a-h, h-a, b-i and i-b), resulting in 12 trials which were randomly repeated five times.

*General processing speed task (control task):* A task similar to the one described by Reigosa-Crespo and colleagues [26] was used. A black square was presented at the center of the screen, and participants were asked to press the space bar as soon as they saw the square. After the response, the square was removed and followed by an interstimulus presentation time varying between 500 and 1500 ms. The test consisted of 20 trials, which were preceded by five practice trials.

#### Standardized tests

*Arithmetical abilities:* Participants were tested with the Tempo Test Arithmetic (TTR) [27]. This timed test consists of five subtests: one for each type of operation (addition, subtraction, multiplication and division) and one with mixed operations. Forty items of increasing complexity were presented in each subtest, and participants were given one minute to solve as many problems as possible. One point was awarded for each correct item.

*Intellectual reasoning (control task)*: Participants completed set II of the Advanced Progressive Matrices (APM) [28] so that their intellectual abilities could be measured. With one point for each correct answer, the maximum score is 36.

## Results

### Experimental tasks

#### General processing speed task

Based on inspection of the reaction time (RT) distribution, cut-offs were determined to eliminate on the one hand anticipatory and on the other very slow responses in such a way that the data loss was maximally 2.5% (i.e. very extreme responses). RTs smaller than 150 ms and larger than 500 ms were excluded from the analysis; this resulted in a 1.15% data loss. Mean RT on this task was 247.95 ms (*SD* =  18.73 ms).

#### Audiovisual matching tasks

Performance was at ceiling in the audiovisual matching tasks (i.e. 97.04%, *SD* = 2.75%; 96.81%, *SD* = 2.47% and 97.17%, *SD* = 2.32% correct for the digit-number word, the dots-number word and the letter-speech sound task respectively). Therefore, we only analyzed the reaction time data.

Mean RTs from correct responses were submitted to a repeated measures analysis of variance (ANOVA) with experimental task (three levels: digits, dots and letters) and numerical distance (three levels: same, different close and different far) as within-subject variables. Following the same procedure as in the general processing speed task, RTs smaller than 250 ms and larger than 1200 ms were excluded from the analysis, resulting in an extra loss of 2.12% of the digit-number word task data, 2.37% of the dots-number word task data, and 2.47% of the letter-speech sound task data (- when the trials with the extreme responses were included in the analyses, the result pattern remained identical).

There was a main effect of numerical distance, *F*(2,50)  = 29.23, *p*<.001, *η_p_^2^* = .54, and a task x numerical distance interaction, *F*(4,48)  = 11.44, *p*<.001, *η_p_^2^* = .49. Pairwise comparisons demonstrated that only in the ‘dots-number word matching task’, the RTs significantly differed between the distances (all *p*'s ≤.01; paired t-test between different small and different large: *t*(51)  = 9.67, *p*<.001); whereas this was not the case for the digit-number word matching task and the letter-speech sound matching task (all *p*'s >.07). Table 1 presents the mean RTs on the three experimental tasks per numerical distance.

**Table 1:** Mean reaction times (and corresponding standard deviations) on the three experimental audiovisual matching tasks per numerical distance.

	Digit-number word matching task	Dots-number word matching task	Letter-speech sound matching task
Distance			
Same	652.78 *(98.75)*	653.66 *(83.30)*	663.03 *(91.03)*
Different close	658.93 *(88.25)*	682.49 *(93.78)*	666.27 *(107.73)*
Different far	648.35 *(100.42)*	631.74 *(90.73)*	669.16 *(103.48)*

### Standardized tests: arithmetical abilities and intellectual reasoning

Participants on average scored 160.31 (*SD* = 21.00) out of 200 on the arithmetic test. Their score on the intellectual reasoning tasks was on average 26.81 (*SD* = 4.56) out of 36.

### Correlation analysis

**Figure 2:**
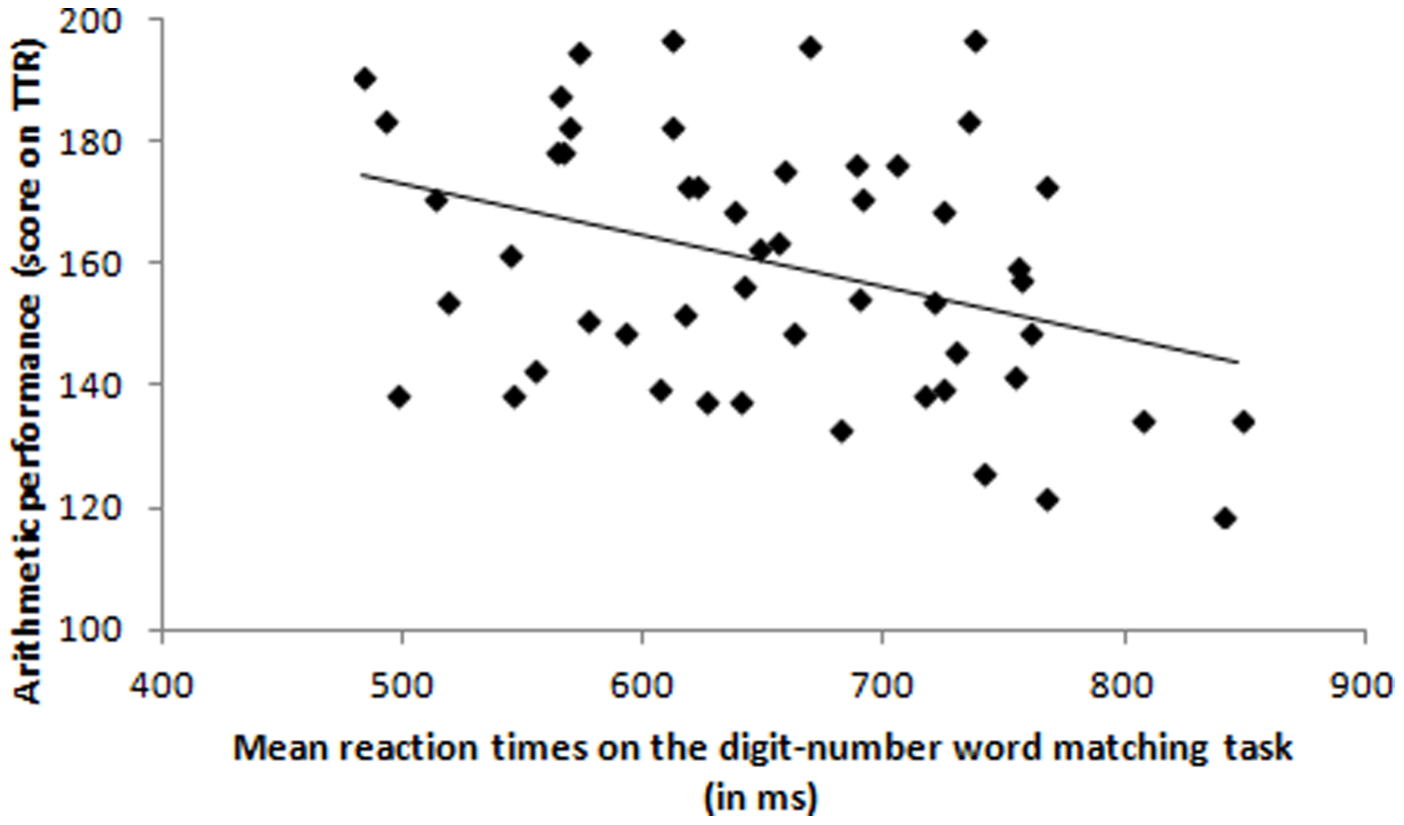
Scatter plot showing a significant correlation between the mean reaction times on the digit-number word matching task and the participants' scores on the standardized arithmetic test (TTR).

The relationship between the participants' reaction times on the audiovisual matching tasks and their scores on the standardized arithmetic test were examined using partial correlations, controlling for general processing speed and intellectual reasoning. Evidently, positive correlations were found between the different audiovisual matching tasks because of their resemblance (all *r_p_*(48) ≥.62). Crucially, there was a marginally significant correlation between the mean RTs on the digit-number word matching task and the standardized arithmetic test scores, *r_p_*(48) = −.36, *p* = .010. Participants who were faster at matching an Arabic digit to the number word they heard obtained higher scores on the standardized arithmetic test (see Figure 2). None of the other correlations were significant, all *r_p_*(48) ≥.12 (see Table 2).

**Table 2:** Partial correlations between the mean reaction times (RT) on the audiovisual matching tasks and the scores on the standardized arithmetic test, controlled for general processing speed and intellectual reasoning (N = 27).

	Arithmetic test score	RT digits-number word matching	RT dots-number word matching
**RT digit-number word matching**	−.36**		
**RT dots-number word matching**	−.16	.75***	
**RT letter-speech sound matching**	−.22	.62***	.69***

** *p*≤.01; ***p≤.001

### Regression analysis

To further explore the unique contribution of performance on the digit-number word matching task to the variance in arithmetic, a hierarchical regression analysis was conducted (see Table 3). In Step 1, the mean scores on the Raven intellectual reasoning test and the mean RTs on the general processing speed task were included to control for general intellectual abilities and general processing speed respectively. In Step 2, mean RTs on the dots-number word matching task and the letter-number word matching task were included to examine whether performance on these tasks contributed to the variance in arithmetic. Finally, in Step 3, the mean RT on the digit-number word matching task was entered into the model to examine the unique contribution of this task to arithmetic abilities, on top of the control measures (intellectual abilities and general processing speed) and on top of the performance on the other two audiovisual matching tasks (dots-number word matching and letter-speech sound matching).

**Table 3:** Hierarchical regression analysis investigating the unique contribution of the performance on the audiovisual matching tasks to the variance in arithmetic achievement.

Step	Independent variables	Standardized *β*	*t*	*p*
1	Raven IQ	.09	.58	.57
	General processing speed	.09	.63	.53
	*F*_change_ <1			
2	Mean RT dot-number word matching task	.33	1.44	.16
	Mean RT letter-speech sound matching task	−.13	−.63	.53
	*F*_change_ (2,47) = 1.24, *p* = .30, *R^2^* = .05			
3	Mean RT digit-number word matching task	−.57	−2.57	.01
	*F*_change_ (1,46) = 6.58, *p* = .01, *R^2^* = .12			

*Note.* Standardized betas from the last step in the analyses are displayed.

The results revealed that only performance on the digit-number word matching task significantly contributed to the model, *β* = −.57, *p* = .014. In total, the model explained 18% of the variance in arithmetic. In contrast, dots-number word matching performance and letter-speech sound matching performance did not contribute to arithmetic achievement when entered into the model after controlling for the other tasks, *β* = .33, *p* = .16 and *β* = −.13, *p* = .53 respectively.

## Discussion

In this study, we wanted to distinguish between (1) the hypothesis that arithmetic achievement is related to performance on symbolic number tasks because the non-symbolic magnitude representation is *accessed* when people are confronted with symbols, and (2) the hypothesis that arithmetic builds on *fast and automatic processing of symbols* (i.e. without the underlying non-symbolic magnitude representation being activated). To address this question, adult participants performed an audiovisual matching paradigm in which auditory presented number words needed to be matched with either Arabic digits or dot patterns. The results revealed that only performance on the digit-number word matching task significantly contributed to the variance in arithmetic. Also, no distance effect (DE) was present in this digit-number word matching task, indicating that the magnitude representation was not addressed and that processing numerical symbols might thus rely on a specialized circuitry for numeric symbol processing [29]. Finally, the absence of a correlation between arithmetic achievement and performance on the letter-speech sound matching task suggested that the relation between symbol processing and arithmetic achievement is number-specific.

What do these results mean with respect to the different theories of numerical cognition? The currently most popular theory remains the Triple Code Model of Dehaene and Cohen [30]. This theory postulates three main number representations – a visual Arabic code in which numbers are represented as Arabic digits (e.g. ‘6’), a magnitude code in which numbers are represented as analogue magnitudes (e.g. ‘••••••’); and a verbal code in which numbers are represented as number words (e.g. ‘six’). These three representations are assumed to be bidirectionally interconnected in the left hemisphere and to include a direct asemantic route for transcoding between Arabic and verbal representations. In the right hemisphere, bidirectional routes are present between the Arabic and magnitude code, but no verbal representation of numbers exists. In terms of this model, the results of the current study might suggest that adults' arithmetical abilities build on the asemantic transcoding route between Arabic and verbal representations, instead of the Arabic magnitude representation circuitry, as hypothesized by Rousselle and Noël [7]. However, this idea is not entirely compatible with the study of Defever and colleagues [31], who found that visual matching of Arabic digits with dot patterns is related to mathematics achievement. Still, it must be noted that the common factor in the digit-number word matching task used in our study and the visual digit-dot matching task conducted in the Defever et al.'s study [31] is the processing of Arabic digits. Both findings can thus be reconciled by assuming that *fast and automatic processing of Arabic digits* is associated with arithmetic. It consequently also becomes clear that the hypothesis of ‘an association between arithmetic and the fast processing of symbols per se’ is particularly of importance to Arabic digits, and not to other symbols in general.

It must be noted that we explicitly chose to only include numerosities from within the subitizing range [24] in the dots-number word matching task to ensure that participants could rapidly identify the presented magnitude. However, since it has been hypothesized that people might process small and large numerosities using two different systems [32], it is unclear whether our results would also account for larger numerosities. It should also be stressed that only adults were focused on in this study and that it remains unclear whether similar conclusions could be drawn for children. Future developmental studies might examine the hypothesis that fast and automatic processing of Arabic digits without the underlying non-symbolic magnitude being activated underlies arithmetic achievement, and also examine whether this relationship changes over time. For these studies, the audiovisual matching paradigm used in the present study would be ideally suited, and this for two reasons. First, the physical similarity between the stimuli and other visual stimulus properties would not play a role in this paradigm as they sometimes do in studies using two visual stimuli [29], [31], [33]. Second, the audiovisual paradigm can be easily used with young children because it does not require them to be fluent readers (this in contrast to when written number words are used).

In sum, we can thus conclude that individual arithmetic differences are associated with the ability to quickly and automatically process Arabic digits without the magnitude representation being activated. In line with this, Cirino [34] already demonstrated that basic digits tasks like identification and rapid naming are related to mathematics achievement. Other studies, however, observed a clear relation between the symbolic DE and mathematics achievement [35], [2], or revealed an association between mathematics achievement and symbolic comparison, even after they controlled for digit identification [36], [37]. Together, these findings emphasize that further research is necessary to pinpoint which steps in the ‘symbol processing chain’ explain the variance in arithmetic.
